# TMT Proteomics-Based Study of Proteins and Pathways Associated with β-Glucan Degradation in Barley Germination

**DOI:** 10.3390/metabo16040250

**Published:** 2026-04-07

**Authors:** Jie Huang, Fangfang Ning, Guoqiang Zhang

**Affiliations:** 1 School of Biological and Food Engineering, Anhui Polytechnic University, Wuhu 241000, China; huangjie1838148@163.com; 2 Wuhu Green Food Industrial Research Institute Co., Ltd., Wuhu 241000, China; nf463590881@163.com

**Keywords:** β-glucan, zangqing ‘1127’, proteomics, seed germination, TMT

## Abstract

**Background**: Zangqing ‘1127’, a hull-less barley type recognized for its high β-glucan content, holds significant agricultural and nutritional potential. Nonetheless, the molecular mechanisms underlying the degradation of β-glucan during barley germination have yet to be thoroughly investigated. **Objectives**: This study sought to identify the key proteins and pathways involved in this process using quantitative proteomics. **Methods**: Seeds of Zangqing ‘1127’ were collected at 0, 24, and 96 h post germination, and TMT-based quantitative proteomics was used to analyze changes in the proteome. To annotate the functions of differentially expressed proteins (DEPs), Gene Ontology (GO) and Kyoto Encyclopedia of Genes and Genomes (KEGG) enrichment analyses were performed. **Results**: In total, 3230 unique proteins were identified, which included 610 DEPs during the germination phase. Enrichment analysis showed that these DEPs were primarily associated with key biological processes involved in β-glucan degradation, including cell wall modification, polysaccharide metabolism, and carbon metabolism. Five proteins exhibiting notably high expression levels were identified as potential regulatory candidates for this process. **Conclusions**: These results enhance our comprehension of the proteomic dynamics associated with β-glucan degradation during barley germination and suggest new candidate targets for functional studies. This study provides deeper insight into the molecular mechanisms governing β-glucan metabolism, with potential implications for agricultural improvement and the nutritional quality of barley.

## 1. Introduction

Barley (*Hordeum vulgare*) is an adaptable crop known for its ability to thrive in harsh and cold environments [1]. It is the fourth most significant cereal grain worldwide, only behind wheat, maize, and rice [2], and boasts a long history of cultivation [3]. Hull-less barley, often referred to as naked barley, is a variety that has been cultivated from common barley. Because of its advantageous processing properties, hull-less barley is vital for human dietary needs and is especially appreciated as a whole-grain option [4]. In addition, hull-less barley is essential for producing animal feed and for malt brewing [2]. Barley β-glucan has also been reported to provide health benefits, particularly in lowering blood glucose levels [5]. Recently, this variety has attracted considerable research attention due to its higher β-glucan content compared to other cereals [4,5,6,7,8]. Importantly, the Zangqing ‘1127’ variety, known for its high β-glucan concentration in grains, has become a major crop widely cultivated in Xizang, China [8,9].

β-Glucan is a type of hemicellulose and serves as a key component of total dietary fiber in barley, together with arabinoxylan [6]. This unique polysaccharide is a linear, homogeneous non-starch polymer composed of D-glucopyranosyl units linked by β-1,3- and β-1,4-glycosidic bonds [10,11]. Two isomers of β-glucan exist, β-D-(1 → 4) glucan and β-D-(1 → 3) glucan, both of which have substantial physiological functions and health benefits [11]. Studies indicate that a large proportion of β-glucan is located in the cell walls of barley grains. Approximately 70% of (1,3;1,4)-β-glucan is found in the cell walls of the starchy endosperm, while about 20% is present in the cell walls of the aleurone layer [4,7,12]. Notably, during germination, β-glucan acts as the main storage substance in the barley cell wall and is degraded by specific hydrolytic enzymes [13,14,15]. This catabolic process actively converts β-glucan into bioavailable forms that are readily absorbed and utilized by the seed, thereby providing essential nutrients and energy for seedling growth and development [16]. In addition, a significant reduction in β-glucan content has been observed during germination [14], further highlighting its critical role as an energy source during barley germination.

Recent advances in genomic technologies have enabled researchers to obtain extensive datasets related to the barley genome, greatly promoting research progress in this field [11]. Early studies have provided comprehensive analyses of gene expression regulation during barley seed development and germination. The regulatory role of the *CslF6* gene in β-glucan content has been well documented [12,17,18]. Further research has shown that isoenzymes EI and EII, encoded by GlbI and GlbII, specifically target the (1 → 4 linkages in (1,3;1,4)-β-glucan. These specific (1,3;1,4)-β-glucanases are critical for normal development and germination of barley seeds [19,20]. In barley, two genes—*HvGlbI* and *HvGlbII*—encoding (1,3;1,4)-β-glucan endohydrolases show elevated expression in the endosperm, roots, and aleurone layer during germination [5]. Through detailed analysis of quantitative trait locus (QTL) mapping and genome-wide association studies (GWASs), researchers have identified a glucan endo-1,3-β-glucosidase gene located near specific single-nucleotide polymorphism (SNP) markers that is closely associated with Bg QTL 9. In addition, three other glucan endo-1,3-β-glucosidase genes have been mapped within Hvs_Bg QTL 1, 4, and 5, all of which contribute to the hydrolysis of glycosidic bonds in β-glucan [7].

Although extensive studies have explored the molecular mechanisms underlying β-glucan synthesis and accumulation in barley [7,8], critical knowledge gaps remain regarding the pathways involved in its degradation and metabolism. In particular, information on the proteins and enzymes involved in β-glucan catabolism in hull-less barley grains remains limited [8]. Therefore, this study used tandem mass tag (TMT)-based quantitative proteomics to investigate proteomic changes associated with β-glucan degradation during germination of the hull-less barley cultivar Zangqing ‘1127’. This study provides new insights into β-glucan metabolism, advancing our understanding of barley growth and development while supporting the breeding of high-yield, high-quality hull-less barley varieties. Previous studies have demonstrated that extracts from germinated barley possess antioxidant activity and antihypertensive effects [21,22,23]. This research also provides a foundation for the development of germinated barley as a functional food source.

## 2. Materials and Methods

### 2.1. Plant Materials and Growth Conditions

**Figure 1:**
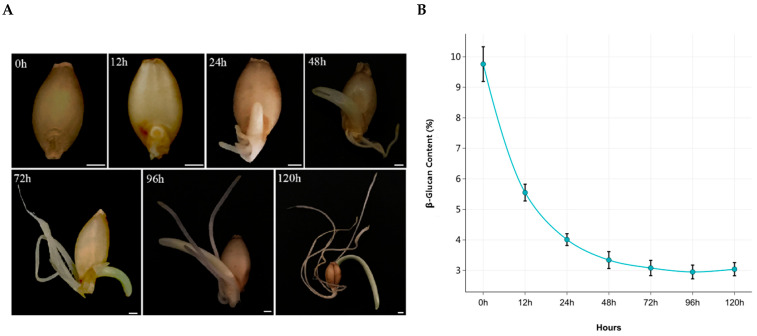
Changes in the germination process and β-glucan content of barley seeds. (**A**) Phenotypes of barley seeds germinated for 120 h. (**B**) Changes in β-glucan content during germination (β-glucan content was measured at 0, 12, 24, 48, 72, 96, and 120 h after imbibition. Data are presented as mean ± standard deviation (SD) of three biological replicates.).

Seeds of the hull-less barley cultivar Zangqing ‘1127’, which is characterized by high β-glucan content, were obtained from the Tibetan Academy of Agricultural and Animal Husbandry. The seeds were surface-sterilized with 1% sodium hypochlorite solution for 15 min and rinsed thoroughly with distilled water. After sterilization, the seeds were soaked in distilled water at 25 °C for 10 h. The soaked seeds were then placed in germination containers with 600 mL of distilled water and germinated at 25 °C and 80% relative humidity for 5 days, with the water renewed daily, using a constant temperature and humidity incubator (LHP-160, Shanghai Sanfa Scientific Instrument Co., Ltd., Shanghai, China). Based on the dynamic changes in β-glucan content during germination (Figure 1B), three representative time points were selected: 0 h (baseline), 24 h (rapid degradation phase), and 96 h (degradation plateau), consistent with previous observations in high-β-glucan barley varieties [14] (Figure 1B). Three biological replicates were collected at each time point, immediately frozen in liquid nitrogen, and stored at −80 °C for subsequent TMT-based proteomic analysis.

### 2.2. Determination of β-Glucan Contents

To maintain the integrity and stability of barley samples, the materials were first snap-frozen in liquid nitrogen. The samples were then finely ground under cryogenic conditions using a Retch M400 ball mill (Retsch GmbH, Haan, Germany) to ensure uniform particle size and complete cell disruption. The β-glucan content was determined using a β-glucan assay kit (Megazyme, Bray, Ireland) following the methods described in previous studies [7,13]. For mixed-linkage β-glucan analysis, exactly 100 mg of the ground barley powder was weighed. All measurements were performed in triplicate to ensure the accuracy and reliability of the data.

### 2.3. Sample Processing

Pulverized tissues were transferred from liquid nitrogen into 5 mL Eppendorf (EP) tubes (Shanghai RonMark, China). For protein extraction, 1000 µL of sodium deoxycholate–Tris (SDT) lysis buffer (4% SDS, 100 mM Tris-HCl, 100 mM DTT, pH 8.0) was added to each tube [24]. The mixture was heated at 100 °C for 5 min and then sonicated on ice using an ultrasonic cell disruptor (JY92-IIDN, Scientz Biotechnology Co., Ltd., Ningbo, China) (25 W, 3 s on, 7 s off) for 5 min. Subsequently, the lysates were centrifuged at 14,000× *g* for 30 min, and the supernatant was filtered through a 0.22 µm ultrafiltration tube(Shanghai BKMAM, China). The protein solution was stored at −80 °C until further analysis2.4. BCA Quantification and SDS-PAGE

Protein concentration was determined using a bicinchoninic acid (BCA) assay kit (Thermo Scientific, Waltham, MA, USA) according to the manufacturer’s instructions. For quality control, 20 µg of protein from each sample was separated by sodium dodecyl sulfate–polyacrylamide gel electrophoresis (SDS-PAGE) and visualized using Coomassie Blue staining.

### 2.4. Enzymatic Digestion

An internal standard (IS) was prepared by mixing 100 µg of protein from each sample. For digestion, 300 µg of protein from each sample and the IS were processed using the Filter-Aided Sample Preparation (FASP) method. Briefly, samples were washed three times with 200 µL of urea (UA) buffer (8 M urea, 150 mM Tris-HCl, pH 8.5) by centrifugation at 14,000× *g* for 30 min at room temperature using a refrigerated microcentrifuge (D3024R, Dalong Xingchuang Experimental Instrument Co., Ltd., Beijing, China). For reduction and alkylation, samples were incubated with 50 mM iodoacetamide (IAA) in UA buffer for 30 min in the dark at room temperature, followed by three washes with UA buffer. Subsequently, samples were washed three times with 100 µL of sodium deoxycholate (DS) buffer (100 mM) under the same centrifugation conditions. Proteins were digested with 52 µL of trypsin buffer (6 µg trypsin in 40 µL DS buffer) at 37 °C for 18 h with shaking at 300 rpm using a thermomixer (ThermoMixer C with interchangeable heating blocks, Eppendorf, Hamburg, Germany). The resulting peptides were collected by centrifugation at 14,000× *g* for 30 min, eluted with an additional 40 µL of DS buffer, and centrifuged again. Peptide concentration was determined by measuring absorbance at 280 nm (OD_280_) using an Enzyme-linked immunosorbent assay reader (SPECTRAMAX M5, Thermo Fisher Scientific, Shanghai, China) [24].

### 2.5. Peptide Labeling and HpH Fractionation

Peptides (100 µg per sample) were labeled using the TMT10plex™ Isobaric Mass Tagging Kit (Thermo Scientific) according to the manufacturer’s protocol. The labeled peptides were mixed and fractionated using high-pH reverse-phase high-performance liquid chromatography (HpH-RP HPLC) on a Gemini-NX 4.6 × 150 mm column (3 µm, 110 Å; Phenomenex Inc., Torrance, CA, USA). Separation was performed using a Dionex UltiMate 3000 system (Thermo Scientific, Waltham, MA, USA) with a gradient composed of Buffer A (10 mM ammonium acetate, pH 10.0) and Buffer B (10 mM ammonium acetate with 90% acetonitrile, pH 10.0). A total of 30 fractions were collected and combined into 10 pools according to their chromatographic profiles. The pooled fractions were freeze-dried and stored at −80 °C until analysis [25].

### 2.6. Liquid Chromatography−Tandem Mass Spectrometry (LC-MS/MS)

Peptides were separated on a Thermo Scientific C18 analytical column (75 µm × 25 cm, 5 µm, 100 Å) using a 60 min gradient at a flow rate of 300 nL/min. Mobile phase A was 0.1% formic acid in water, and mobile phase B was 0.1% formic acid in acetonitrile. The gradient was set as follows: 5% to 28% B over 40 min, 28% to 90% B over 2 min, and maintained at 90% B for 18 min. Samples were first loaded onto a Thermo Scientific EASY trap column (100 µm × 2 cm, 5 µm, 100 Å) for desalting. Eluted peptides were analyzed using an Orbitrap Fusion mass spectrometer (Thermo Scientific, Waltham, MA, USA) in positive ion mode with a full scan range of 375–1800 *m*/*z*.

The Orbitrap Fusion was operated in data-dependent acquisition (DDA) mode. Full-scan MS spectra were acquired over 375–1800 *m*/*z* at a resolution of 120,000 (*m*/*z* 200), with an automatic gain control (AGC) target of 4 × 10^5^ and a maximum injection time of 50 ms. For MS^2^ analysis, precursors were selected in Top-Speed mode with a 3 s cycle time, and dynamic exclusion was set to 40 s. Higher-energy collisional dissociation (HCD) fragmentation was performed, and MS^2^ spectra were acquired at a resolution of 50,000 (*m*/*z* 200), with an AGC target of 1 × 10^5^, a maximum injection time of 105 ms, and 1 microscan.

### 2.7. Mass Spectrometry Data Analysis

Raw mass spectrometry data were analyzed using Proteome Discoverer 2.1 (Thermo Scientific, Waltham, MA, USA) with the Mascot 2.3 search engine against the UniProt barley database (uniprot_domesticated_barley_207518_20190314.fasta, 207,518 sequences). Peptides were identified with a false discovery rate (FDR) ≤ 0.01. Protein quantification was based on peptide ion peak intensities, and protein levels were calculated as the median of corresponding peptide quantifications. Median normalization across all channels was performed to reduce experimental variation.

### 2.8. Bioinformatics Analysis

Gene Ontology (GO) enrichment analyses were conducted using the DAVID 6.7 database (https://davidbioinformatics.nih.gov/) [26] accessed on 2023 and QuickGO (https://www.ebi.ac.uk/QuickGO/) [27]. GO terms were categorized into three distinct classifications: biological process (BP), cellular component (CC), and molecular function (MF). KEGG pathway enrichment analysis was performed using the Kyoto Encyclopedia of Genes and Genomes (KEGG) database (https://www.kegg.jp/) [28]. For both GO and KEGG analyses, enrichment significance was assessed using Fisher’s exact test, with a significance threshold set at *p* < 0.05. All quantified proteins served as the background reference. Protein–protein interaction (PPI) network analysis was performed using the STRING database (https://string-db.org/) [29] and visualized with Cytoscape software (https://www.cytoscape.org/) [30].

### 2.9. Real-Time Fluorescence Quantitative PCR (qPCR)

Total RNA was extracted from samples A, B, and C using the RNApure Kit (DP430, Tiangen Biotech (Beijing) Co., Ltd., Beijing, China) according to the manufacturer’s protocol. First-strand cDNA was synthesized using the PrimeScript RT Reagent Kit with gDNA Eraser (Takara, Dalian, China). qPCR was performed in a 20 µL reaction mixture containing 2 µL of cDNA, 0.5 µL each of forward and reverse primers (10 µmol/L), 10 µL of qPCR SuperMix, and 7 µL of ddH_2_O. The thermal cycling program was as follows: 94 °C for 30 s; 41 cycles of 94 °C for 5 s and 60 °C for 32 s using a CFX96 Real-Time PCR system (Bio-Rad Laboratories, Inc., Hercules, CA, USA). Melting curve analysis was performed after amplification. Gene expression levels were calculated using the 2^−ΔΔCt^ method with *Hv_actin* as the internal reference gene. Primer sequences are listed in Table S1. All reactions were performed in triplicate.

### 2.10. Statistical Analysis

Experimental data were statistically analyzed using Statistical Package for the Social Sciences (SPSS) 27.0 (IBM, Armonk, NY, USA). Dunnett’s test was used to evaluate significant differences between groups, with *p* < 0.05 considered statistically significant. All measurements were performed at least in triplicate, and the results are expressed as the mean ± standard error of the mean (SEM).

## 3. Results

### 3.1. Morphological Changes and β-Glucan Levels in Barley Seeds

Figure 1A presents the morphological characteristics of barley seeds at various stages of germination. As the germination process advanced, the roots and shoots of these seeds exhibited consistent growth. At the same time, the β-glucan content demonstrated a recognizable pattern: a pronounced decrease occurred within the first 24 h of germination, followed by a gradual stabilization of this downward trend. By the time 96 h had passed, the β-glucan content had reached its lowest point, only to show a slight rise thereafter (Figure 1B). This observed trend is consistent with previous studies [31,32,33], which corroborate the breakdown of β-glucan in barley during the germination phase. Statistical evaluation (Figure S1) revealed significant differences (*p* < 0.05) in protein expression among samples A, B, and C, indicating distinct protein profiles across germination stages.

In summary, these results demonstrate that the degradation of β-glucan occurs primarily during the first 24 h of germination, accompanied by distinct shifts in protein expression profiles that differentiate the three sampled time points.

### 3.2. TMT Analysis of Protein Expression Between B vs. A, C vs. B, and C vs. A

By establishing a screening threshold of peptide FDR ≤ 0.01, we identified 3230 proteins and 14,285 peptides (Table S2). To improve data quality, we further filtered the identified proteins, retaining only those with quantitative information across all 10 labeled channels, resulting in a final set of 3168 proteins. These proteins were employed to assess reporter ion intensities and investigate variations in the differential proteome across three distinct time points. Pearson correlation analysis revealed high reproducibility among biological replicates, with correlation coefficients r > 0.95 for all samples (Figure S2), confirming the reliability of the protein quantification.

**Figure 2:**
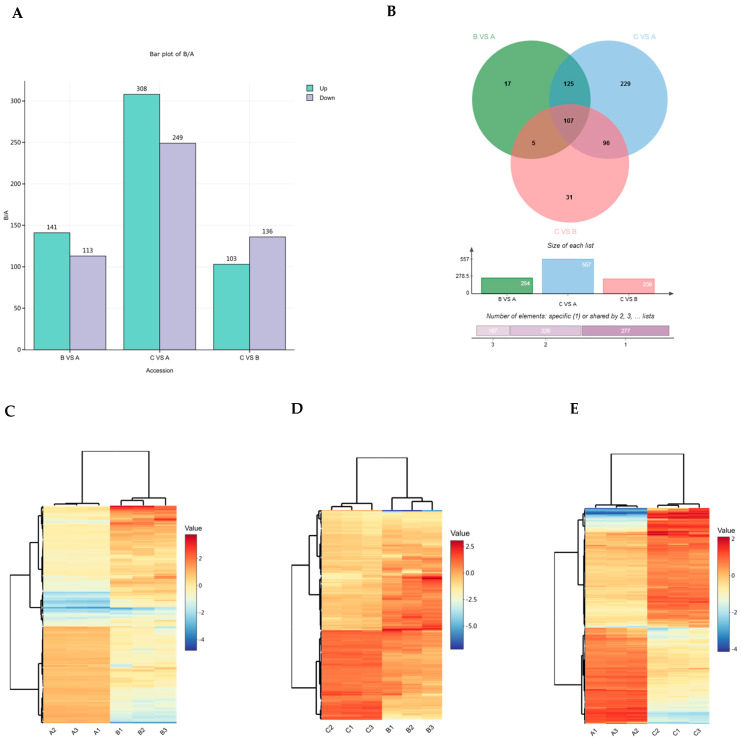
An overview of differentially expressed proteins (DEPs) identified in the three comparisons (B vs. A, C vs. B, and C vs. A). (**A**) A bar graph showing the numbers of upregulated (cyan) and downregulated (purple) DEPs in each comparison. (**B**) A Venn diagram illustrating the overlap of DEPs among the three comparisons. (**C**–**E**) Heat maps showing hierarchical clustering of DEPs in (**C**) B vs. A, (**D**) C vs. B, and (**E**) C vs. A. The color scale represents log_2_-transformed relative expression levels, with orange to red indicating upregulated proteins and blue indicating downregulated proteins. Samples A, B, and C correspond to 0, 24, and 96 h after imbibition, respectively.

Differentially expressed proteins (DEPs) were identified using a threshold of *p* < 0.05 and |log_2_FC| > log_2_(1.2) (i.e., fold change > 1.2) in the three pairwise comparisons (B vs. A, C vs. B, and C vs. A). A total of 610 DEPs were identified across the three comparisons (Table S3). The numbers of up- and downregulated DEPs in each comparison are summarized in Figure 2A,B.

Volcano plots and heat maps of differentially expressed proteins (DEPs) identified in the three comparisons (B vs. A, C vs. B, and C vs. A) are shown in Figure 2C–E and Figure S3. The B vs. A comparison revealed 254 DEPs, including 141 upregulated and 113 downregulated proteins (Figure 2C). In the C vs. B comparison, 239 DEPs were identified, with 103 upregulated and 136 downregulated (Figure 2D). The C vs. A comparison yielded 557 DEPs, comprising 308 upregulated and 249 downregulated proteins (Figure 2E). Overlap analysis revealed both shared and unique DEPs among the three comparisons (Figure 2B). Hierarchical clustering of all DEPs showed distinct expression patterns across the germination stages (Figure 2C–E; Table S4), facilitating the identification of candidate proteins potentially involved in β-glucan degradation.

In summary, a total of 3168 high-confidence proteins were quantified with excellent reproducibility (r > 0.95) across biological replicates. Among these, 610 DEPs were identified across the three germination stages, with the largest number of changes observed between 0 and 24 h (254 DEPs), followed by the transition from 24 to 96 h (239 DEPs), and the overall comparison between 0 and 96 h (557 DEPs). Overlap analysis revealed stage-specific and shared DEPs, while hierarchical clustering highlighted distinct expression patterns that correlate with the dynamics of β-glucan degradation during germination.

### 3.3. Identification of Differentially Abundant Proteins

**Figure 3:**
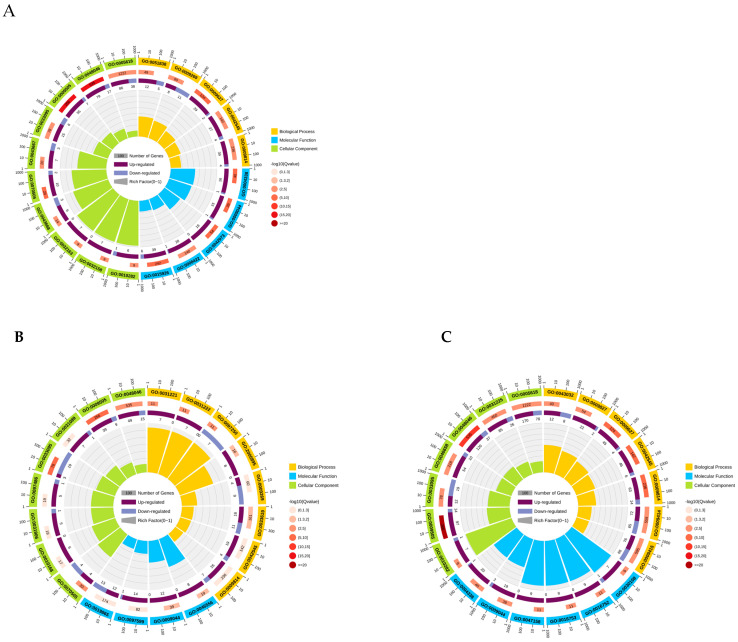
GO enrichment analysis of differentially expressed proteins (DEPs). (**A**–**C**) GO term enrichment (biological process, BP; molecular function, MF; cellular component, CC) for the comparisons (**A**) B vs. A (24 h vs. 0 h), (**B**) C vs. B (96 h vs. 24 h), and (**C**) C vs. A (96 h vs. 0 h). The inner ring indicates the number of upregulated (dark purple) and downregulated (light purple) DEPs; the outer ring color represents the significance of enrichment, expressed as –log_10_(*Q*-value). Yellow, blue, and green denote BP, MF, and CC categories, respectively.

GO annotation of the DEPs is summarized in Figure S4 and Table S5. The DEPs were classified into three main categories: biological process (BP), cellular component (CC), and molecular function (MF). The distribution of GO terms across the three comparisons (B vs. A, C vs. B, and C vs. A) is shown in Figure S4A–C. The numbers of GO terms with increased or decreased expression in each comparison are detailed in Table S6. GO enrichment analysis of DEPs was performed for the three comparisons (B vs. A, C vs. B, and C vs. A) with a significance threshold of *p* < 0.01 (Table S6). In the B vs. A comparison, DEPs were significantly enriched in cellular components associated with the apoplast, plant-type cell wall, aleurone grain, and cell wall structure (Figure 3A). For molecular function, the enriched DEPs were mainly associated with hydrolase activities, including glucan exo-1,3-β-glucosidase, glucosidase, xylan 1,4-β-xylosidase, glucan endo-1,3-β-D-glucosidase, and β-glucosidase. In the biological process category, enriched DEPs were primarily involved in systemic acquired resistance, defense responses, incompatible interactions, cell wall modification, and responses to dehydration and cytolysis (Figure 3A). In the C vs. B comparison, DEPs enriched in the cellular component category were primarily localized to the cytoplasm and plant-type cell wall (Figure 3B). Proteins associated with dextran grains were reduced in this comparison. For molecular function, enriched DEPs were mainly associated with xylan 1,4-β-xylosidase and α-L-arabinofuranosidase activities, while proteins linked to immunoglobulin binding were decreased. In the biological process category, enriched DEPs were involved in arabinan metabolism, arabinan degradation, hemicellulose breakdown, membrane protein degradation, defense responses, incompatible interactions, and cell wall modification. Proteins with negative enrichment were associated with mitochondrial respiration, supercomplex assembly, and responses to dehydration (Figure 3B). In the C vs. A comparison, DEPs enriched in the cellular component category were localized to the apoplast, plant cell wall, and cell wall. Proteins anchored to or associated with the plasma membrane also showed increased levels (Figure 3C). For molecular function, enriched DEPs were mainly associated with xylan 1,4-β-xylosidase and glucan exo-1,3-β-glucosidase activities. In the biological process category, enriched DEPs were involved in defense mechanisms, incompatible interactions, systemic acquired resistance, cell wall modification, and responses to drought conditions (Figure 3C).

In summary, GO enrichment analysis revealed distinct functional profiles across the three germination stages. Consistent with the morphological and β-glucan degradation dynamics observed earlier, DEPs in the early stage (0 h → 24 h, B vs. A) were predominantly enriched in hydrolase activities and cell wall organization components, consistent with active cell wall and β-glucan degradation. In the middle stage (24 h → 96 h, C vs. B), enrichment shifted toward hemicellulose metabolism and defense responses, while the late-stage comparison (0 h → 96 h, C vs. A) showed strong enrichment in defense-related biological processes and cell wall modification. These stage-specific functional profiles provide a proteomic basis for understanding the dynamic regulation of cell wall and β-glucan metabolism during barley germination.

### 3.4. KEGG Pathway Enrichment Analysis

**Figure 4:**
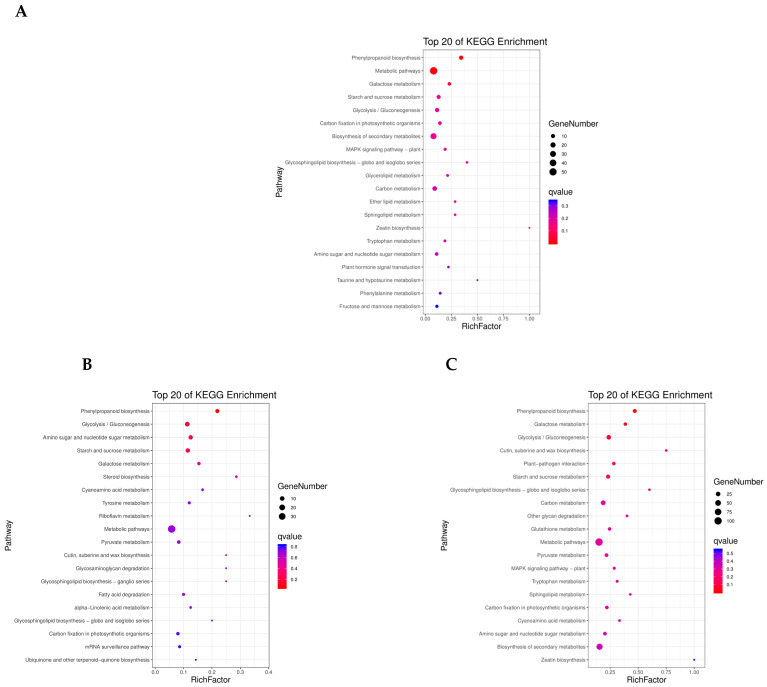
KEGG pathway enrichment analysis of differentially expressed proteins (DEPs). (**A**) B vs. A, (**B**) C vs. B, and (**C**) C vs. A. Bubble size represents the number of proteins enriched in each pathway; bubble color indicates the significance of enrichment, with redder colors corresponding to lower Q-values (higher significance).

KEGG pathway analysis of the DEPs across the three comparisons (B vs. A, C vs. B, and C vs. A) is summarized in Figure S5. The identified pathways were classified into five main categories: metabolism, genetic information processing, environmental information processing, cellular processes, and organismal systems. In the B vs. A comparison, DEPs were enriched in metabolic pathways, phenylpropanoid biosynthesis, and galactose metabolism (Figure 4A). In the C vs. B comparison, enriched pathways included glycolysis/gluconeogenesis, starch and sucrose metabolism, amino sugar and nucleotide sugar metabolism, and phenylpropanoid biosynthesis (Figure 4B). In the C vs. A comparison, DEPs were enriched in plant–pathogen interaction and sugar metabolism pathways (Figure 4C). In the B vs. A comparison, additional enriched pathways included the MAPK signaling pathway, plant-related pathways, tryptophan metabolism, carbon metabolism, and fructose and mannose metabolism (Figure 4A). In the C vs. B comparison, enriched pathways included tyrosine metabolism, pyruvate degradation, α-linolenic acid metabolism, and mRNA monitoring (Figure 4B). In the C vs. A comparison, enriched pathways included plant–pathogen interaction and glycan degradation (Figure 4C).

In summary, KEGG enrichment analysis revealed stage-specific metabolic profiles during barley germination. The early stage (B vs. A) was characterized by enrichment in phenylpropanoid biosynthesis and primary carbohydrate metabolism; the middle stage (C vs. B) showed a shift toward glycolysis and nucleotide sugar metabolism; and the late stage (C vs. A) was marked by enrichment in defense-related pathways such as plant–pathogen interaction and glycan degradation.

### 3.5. Protein−Protein Interaction (PPI) Network of DEPs

**Figure 5:**
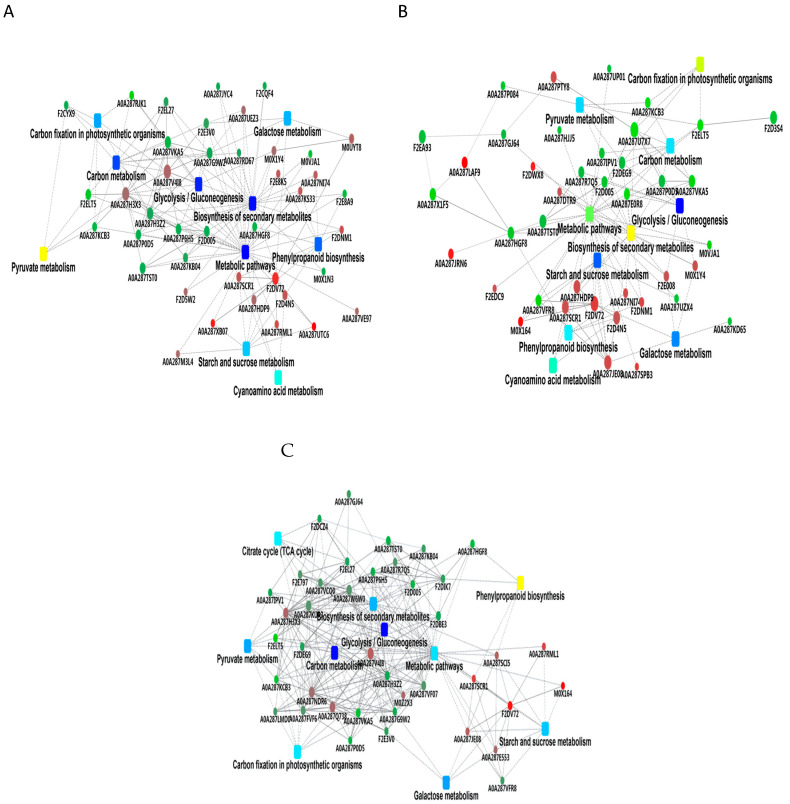
Protein–protein interaction (PPI) networks of differentially expressed proteins (DEPs) focused on the top 10 significantly enriched KEGG pathways. (**A**–**C**) PPI networks for (**A**) B vs. A (24 h vs. 0 h), (**B**) C vs. B (96 h vs. 24 h), and (**C**) C vs. A (96 h vs. 0 h). Nodes: round nodes represent DEPs; rounded rectangles represent KEGG pathways. Node colors: red and green indicate up- and downregulated DEPs, respectively; pathway node colors (yellow to blue gradient) indicate enrichment significance (yellow: low, blue: high).

To investigate the functional relationships among key DEPs potentially involved in β-glucan degradation, we constructed protein–protein interaction (PPI) networks for the B vs. A and C vs. B comparisons (Figure 5A,B). To study the regulatory interactions of DEPs, a global PPI network was constructed using all quantified DEPs (Figure S7A). Within this network, two seed storage proteins—M0XUU4 (FC = 0.54, cupincin) and A0A287MXS1 (FC = 0.53, globulin-like protein)—were identified as interactors localized to the extracellular region and associated with IgE binding. These proteins also interacted with M0XPC4 (FC = 0.62, Os07g0480900 protein), a predicted inhibitor of protein hydrolysis. Additionally, F2CWL7 (FC = 0.49, oleosin) was associated with A0A287P198 (FC = 0.45, late embryogenesis abundant protein 17), and A0A287HGF8 (FC = 0.48, 1-Cys peroxiredoxin A) showed the highest connectivity within the global network.

To focus on pathways relevant to β-glucan degradation and nutrient mobilization, a refined PPI network was constructed based on the 10 most significantly enriched KEGG pathways (Figure 5A). This network highlighted interactions among carbohydrate metabolism enzymes, including F2DV72 (FC = 5.69, a putative α-glucosidase) and M0UYT8 (FC = 2.04, an α-galactosidase), both involved in polysaccharide hydrolysis. Hub proteins within this focused module were associated with starch and sucrose metabolism, glycolysis/gluconeogenesis, and secondary metabolism.

In the C vs. B comparison, the global PPI network (Figure S7B) showed downregulation of several proteins, including M0XUU4 (FC = 0.64), A0A287MXS1 (FC = 0.54), A0A287HGF8 (FC = 0.61), and M0XPC4 (FC = 0.59), consistent with the patterns observed in the B vs. A comparison. F2DEG9 (FC = 0.67, phosphoenolpyruvate carboxykinase) was also found to form interactions within this network. The focused metabolic network (Figure 5B) further localized F2DEG9 within glycolysis/gluconeogenesis and carbon metabolism modules.

For the C vs. A comparison, the global PPI network (Figure S7C) showed that upregulated proteins (red nodes) formed a dense central interaction module, while downregulated proteins (blue nodes) were mainly distributed at the network periphery. The focused metabolic network (Figure 5C) highlighted interactions among key carbohydrate metabolism pathways, including glycolysis/gluconeogenesis, carbon metabolism, and starch and sucrose metabolism. Key enzymes such as F2DEG9 (phosphoenolpyruvate carboxykinase) and F2DV72 (α-glucosidase) acted as central hubs within these modules. Consistent with previous comparisons, seed storage proteins (M0XUU4, A0A287MXS1) and protease inhibitors (M0XPC4) remained downregulated with reduced interaction strength, while A0A287HGF8 maintained high connectivity in the secondary metabolism module.

In summary, PPI network analysis across the three germination stages revealed a consistent downregulation of seed storage proteins (M0XUU4, A0A287MXS1) and protease inhibitors (M0XPC4), while carbohydrate metabolism enzymes (F2DV72, M0UYT8, F2DEG9) formed central hubs in pathways related to starch/sucrose metabolism, glycolysis/gluconeogenesis, and secondary metabolism. These results highlight the dynamic reorganization of protein interaction networks during germination, with a shift from storage mobilization to active carbohydrate metabolism.

### 3.6. Validation in TMT Data by qPCR

**Figure 6:**
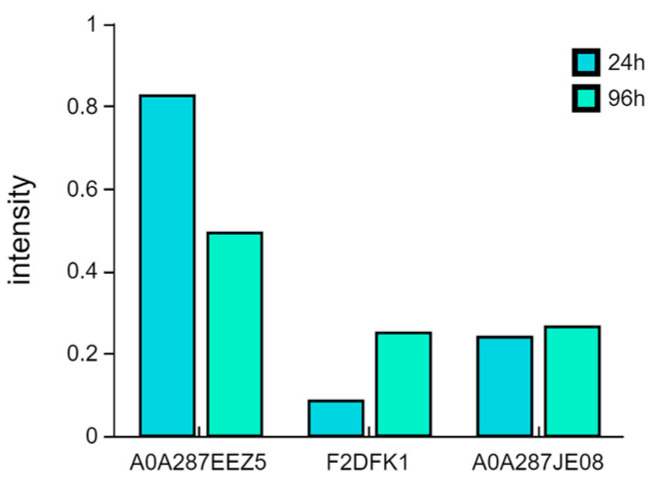
Validation of related proteins involved in the β-glucan degradation pathway in C vs. B by real-time quantitative PCR (qPCR).

To validate the protein expression results obtained by TMT, six differentially expressed proteins (DEPs) closely associated with β-glucan degradation were selected for qPCR analysis (Table S1). In the B vs. A comparison, the expression levels of three DEPs showed minor fluctuations (Figure S6). In the C vs. B comparison, the expression levels of the remaining three DEPs were consistent with the TMT results (Figure 6).

## 4. Discussion

Zangqing ‘1127’ is a hull-less barley variety with high β-glucan content. Previous studies have shown that β-glucan content decreases progressively during barley germination [14,16], and that glucanases such as *C7H5* and *Cel6* play key roles in this process [34]. However, the protein-level mechanisms underlying β-glucan degradation during germination remain poorly understood. In this study, we employed TMT-based quantitative proteomics combined with qPCR validation to investigate the proteins and regulatory pathways involved in β-glucan degradation in hull-less barley.

Seed germination is a dynamic process characterized by various changes, regulated by genetic factors from multiple aspects [35]. In the present study, barley grains exhibited a rapid decrease in β-glucan content within the initial 24 h of germination, followed by a gradual slowdown in degradation rate, reaching the lowest level at 96 h. Notably, a slight re-increase in β-glucan content was observed by 120 h (Figure 1B), which may reflect a dynamic balance between residual degradation and de novo synthesis of mixed-linkage glucan (MLG), consistent with the developmentally regulated MLG deposition pattern reported in cereal grains [36]. To gain mechanistic insights into these dynamic changes, we performed TMT-based quantitative proteomics analysis on samples at 0 h (A), 24 h (B), and 96 h (C) of germination.

A total of 610 uniquely expressed proteins were successfully identified through this analysis. The B vs. A comparison yielded a larger number of DEPs than the C vs. B group, which is consistent with the rapid β-glucan degradation during the early germination stage. The selection of candidate proteins in Table 1 was comprehensively determined by previously documented mechanisms of cereal β-glucan metabolism [8] and the physiological and proteomic data obtained in the present study. The enrichment analyses using GO and KEGG pathways indicated that these proteins were significantly involved in functions such as cell wall modification, cell wall breakdown, polysaccharide metabolism, carbon metabolism, and defense mechanisms [37,38]. The expression patterns and functional evaluations of these proteins offered essential insights into the catabolic processes of β-glucan. A comprehensive discussion of these DEPs will be provided subsequently.

Polysaccharide degradation in endosperm cell walls, particularly β-glucan hydrolysis, is essential for energy supply during barley seed germination [37]. Multiple hydrolytic enzymes identified in the present study contribute to this process, among which β-1,3-glucanase II (Q8S3U1) exhibited distinct upregulation in the early germination stage (B vs. A), while no obvious expression was detected at the subsequent C vs. B stage. This indicates that Q8S3U1 plays a vital role in initiating cell wall polysaccharide breakdown at the early germination phase, directly driving the rapid decline in β-glucan content within 0–24 h.

Glycoside hydrolases are core enzymes responsible for cleaving glycosidic bonds and are indispensable for cell wall polysaccharide and β-glucan degradation during germination. Multiple glycoside hydrolase members with obvious stage-specific expression patterns were identified in our study (Table 1), including mannan endo-1,4-β-mannosidase (F2ECC9, M0YV20) [39], glycosyl hydrolase 17 family proteins (F2EB49, F2DTH1), glycosyl hydrolase 1 family protein (F2D4N5), maltase (F2DV72), and α-galactosidase (M0UYT8). GO functional annotation confirmed that F2EB49, F2D4N5, F2DV72 and F2DTH1 possess glucosidase activity, which is closely associated with plant cell wall remodeling and stress adaptation [40]. Glucosidases synergize with endo-β-glucanase and cellobiose hydrolase to decompose β-glucan and release fermentable glucose, playing a key role in cell wall polysaccharide saccharification [41]. These glycoside hydrolases showed clear temporal expression specificity across germination stages: F2EB49 was predominantly elevated in the early stage (B vs. A), whereas F2DTH1 was specifically up-regulated at the later stage (C vs. B). By contrast, F2D4N5 and F2DV72 were differentially expressed in both comparisons, with far stronger upregulation occurring in the early phase. Mannan endo-1,4-β-mannosidase contributes to degrading cell wall mannose-containing polysaccharides to release soluble carbon sources [39]. F2ECC9 was markedly upregulated in the B vs. A group and facilitates early germination by providing essential energy, while M0YV20 was specifically upregulated in the C vs. B group, participating in cell wall remodeling and sustained polysaccharide metabolism at the late germination stage (Table 1). As a critical exoglycosidase for hemicellulose decomposition, α-galactosidase M0UYT8 was highly expressed in the early germination stage (B vs. A). Consistent with GO annotation linked to cell wall metabolism, M0UYT8 promotes the hydrolysis of galactose-containing cell wall polysaccharides [42], mediates carbohydrate mobilization, and supports energy supply for early barley germination.

Given that β-glucan is a major hemicellulose component in barley cell walls [43], these functionally diverse and spatiotemporally regulated hydrolases coordinately mediate the sequential hydrolysis of cell wall polysaccharides at distinct germination phases, supplying essential carbon sources and energy to support barley seed germination and seedling establishment. Additionally, F2E3V0 (ATP-dependent 6-phosphofructokinase) was significantly downregulated in the B vs. A comparison (Table 1). As a rate-limiting enzyme in the glycolytic pathway, phosphofructokinase catalyzes the irreversible conversion of fructose 6-phosphate to fructose 1,6-bisphosphate and controls the influx of monosaccharides into glycolysis [44,45]. The downregulation of F2E3V0 suggests restrained glycolysis during early barley germination, which may reduce soluble sugar consumption and prioritize the utilization of cell wall-stored carbohydrates such as β-glucan. Moreover, two aldose 1-epimerase proteins were identified (Table 1): M0X1Y4 was upregulated in both B vs. A and C vs. B comparisons, while F2E008 was specifically upregulated in the C vs. B group. Aldose 1-epimerase is a key enzyme mediating the interconversion of α- and β-aldose isomers during carbohydrate metabolism [46]. Elevated expression of this enzyme is closely associated with cell wall remodeling and polysaccharide degradation, coordinating carbon metabolic reprogramming to support continuous energy supply for germination [47].

In addition to β-glucan, starch serves as a critical reserve carbohydrate providing energy for barley seed germination [10]. KEGG enrichment analysis revealed that starch and sucrose metabolic pathways are closely coupled to β-glucan turnover, implying that starch catabolism-related proteins may directly or indirectly modulate β-glucan degradation [48]. Two key starch-degrading enzymes were identified from the proteomic data (Table 1): α-amylase (A0A8I7BF21) was significantly upregulated, while β-amylase (F2DY58) was downregulated in the C vs. B comparison. α-Amylase catalyzes the cleavage of α-1,4-glycosidic bonds in starch to produce soluble small-molecule sugars [49], and it contributes to releasing soluble glucan fragments during cereal germination [50]. The upregulation of A0A8I7BF21 in the late germination stage suggests enhanced starch hydrolysis to supply carbon and energy, which may reduce the dependency on β-glucan consumption at this phase. β-Amylase mainly hydrolyzes α-1,4-glycosidic bonds at the non-reducing ends of polyglucan chains to generate maltose, and distinct subcellular-localized β-amylases function diversely in glucan metabolism [51]. Differing from the previously reported β-amylase (Q6SNP7) associated with elevated β-glucan accumulation in barley [8], the downregulated expression of F2DY58 (β-amylase) in the present study is speculated to facilitate β-glucan degradation. Notably, a previous study on barley reported that overexpression of endogenous (1,3;1,4)-β-D-glucanase isoenzyme EII significantly decreased grain β-glucan content while simultaneously increasing starch levels [52], supporting the reciprocal regulatory relationship between β-glucan degradation and starch metabolism observed in our study. These findings are consistent with earlier reports indicating a coordinated interplay between β-glucan and starch metabolism during cereal grain development [8,53]. During germination, barley seeds activate sophisticated defense systems to cope with biotic and abiotic stresses, enabling environmental adaptation and stress resistance [54,55]. Soluble sugars serve not only as primary energy substrates for respiratory metabolism and defensive processes but also as carbon skeletons for synthesizing defensive secondary metabolites (e.g., flavonoids, lignin) [56]. Furthermore, sugars such as glucose and sucrose act as critical signaling molecules to modulate the expression of defense-related genes. Notably, (1,3;1,4)-β-glucan, the major cell wall polysaccharide in barley endosperm, acts as an important endogenous signaling elicitor during germination [54,55,56,57,58]. In the present proteomic analysis, numerous stress- and defense-related DEPs were identified (Table 1), including peroxidases and chitinases, which were differentially expressed across germination stages. Mechanistically, the continuous degradation of cell wall β-glucan releases soluble sugars and cell wall-derived signaling fragments during seed germination; these products function as endogenic triggers to initiate and amplify plant defense pathways, rather than merely representing passive byproducts of metabolic stress [59,60]. The coordinated turnover of β-glucan and reprogramming of carbohydrate metabolism therefore provide both energy resources and signaling cues, synchronously driving cell wall reserve mobilization, seed growth, and the activation of defense networks to enhance stress tolerance during barley germination. KEGG enrichment analysis indicated that β-glucan degradation during barley germination is closely coupled with the phenylpropanoid biosynthesis pathway [60,61]. Phenylpropanoid-derived metabolites contribute substantially to plant tolerance against abiotic stresses (high temperature, salinity) and biotic pathogen challenges, relying on a series of key catalytic enzymes, among which peroxidases are essential for structural modification of phenolic compounds to enhance stress adaptability [62,63]. In the present study, multiple peroxidase-related DEPs exhibited distinct stage-specific expression patterns during germination (Table 1): catalase (F2DNM1) was upregulated in both B vs. A and C vs. B comparisons; peroxidase (F2E8A9) was downregulated in B vs. A, while peroxidase (F2E8K5) was specifically upregulated at the early germination stage (B vs. A). GO functional annotation confirmed their predominant involvement in stress response pathways. Beyond peroxide scavenging, these peroxidases mediate the oxidation of phenolic substrates in the phenylpropanoid cascade, synergistically facilitating barley seed germination and protecting embryos and endosperm from adverse environmental stressors [64].

Moreover, additional defense- and development-related DEPs were identified (Table 1): several GDSL esterases/lipases (F2D5K9, F2DMQ8, M0Z1D4) and gibberellin-regulated protein (F2ELX8) were differentially activated during germination. GDSL esterases/lipases participate in ester and lipid metabolism and function broadly in plant growth regulation and defense response modulation [64,65,66]. As an important pathogenesis-related protein, chitinase targets fungal cell wall chitin to inhibit pathogenic infection, while also positively regulating seed developmental processes [67,68]. Collectively, alongside sequential β-glucan depolymerization and carbohydrate reallocation, the coordinated expression of peroxidases, GDSL esterases/lipases, chitinase, and other defense-related proteins integrates carbon metabolism, phenylpropanoid biosynthesis, and stress defense networks, ensuring robust growth, stress resistance, and normal developmental progression during barley seed germination [8].

In addition to the aforementioned proteins, several late embryogenesis abundant (LEA) proteins with differential expression were identified in our proteomic analysis (Table 1), exhibiting distinct stage-specific downregulation patterns during barley germination. Specifically, LEA protein (F2DG27) was significantly downregulated in the C vs. B comparison, while M0Y565 and M0WI75 were specifically downregulated in the early germination stage (B vs. A). Notably, F2E9C4 showed significant downregulation in both B vs. A and C vs. B comparisons, indicating continuous downregulation throughout the β-glucan degradation process. Previous studies have confirmed that LEA proteins are actively accumulated during β-glucan anabolism in developing barley grains [8,56,69]. In contrast, our results revealed that these LEA members were gradually suppressed during continuous β-glucan depolymerization at different germination stages, implying their dual involvement in both β-glucan synthetic and degradative pathways; LEA proteins may facilitate β-glucan accumulation during seed maturation, while their downregulation contributes to cell wall polysaccharide remobilization upon germination [56]. Additionally, papain-like cysteine proteases (PLCPs) are indispensable regulators of plant immunity and seed developmental metabolism [70,71]. In the present study, the papain-like cysteine protease B4ESF5 was significantly upregulated in the C vs. B group (Table 1), suggesting its vital role in enhancing defense capacity during the late germination phase. Combined with the intrinsic crosstalk between β-glucan hydrolysis and defense pathway activation in barley, B4ESF5 is speculated to coordinate carbohydrate reserve turnover and stress resistance throughout seed germination.

## 5. Conclusions

In this study, we investigated proteins with differential expression (DEPs) that are linked to the breakdown of β-glucan during the germination stage of barley seeds, utilizing quantitative proteomics techniques with TMT. We identified a total of 3230 proteins across three distinct developmental phases, with 610 DEPs specifically observed during the germination of seeds. Analyses performed using gene ontology (GO) and KEGG pathways revealed that the reduction in β-glucan content was largely associated with activities related to cell wall alteration and degradation, polysaccharide metabolism, pathways of carbon metabolism, and defensive mechanisms. The proteins Q8S3U1, F2ECC9, F2D4N5, F2DV72, and A0A8I6WT53 showed elevated expression levels and played a significant role in the degradation of β-glucan, thereby promoting its catabolism. These findings provide new insights into the proteomic regulation of cell wall polysaccharide turnover during barley germination and offer candidate targets for improving barley quality and germination efficiency.

## Figures and Tables

**Table 1:** List of candidate proteins related to barley β-glucan catabolism.

Accession	Description	Log_2_(FoldChange)	
		B vs. A	C vs. B
F2DFK1	Predicted protein belonging to the peptidase S8 family	0.61	0.81
F2DNM1	Peroxidase	1.47	0.81
F2DX18	Predicted protein belonging to the peptidase S8 family	1.71	1.37
F2E8A9	Peroxidase	−1.11	
F2E8K5	Peroxidase	1.23	
F2ECC9	mannan endo-1,4-beta-mannosidase	2.60	
F2EB49	Predicted protein belonging to the glycosyl hydrolase 17 family	0.59	
Q8S3U1	Beta-1,3-glucanase II	2.55	
F2D4N5	Predicted protein belonging to the glycosyl hydrolase 1 family	1.31	0.61
F2DDK8	Glycosyltransferase	−0.73	
F2EHF9	Predicted protein belonging to the peptidase C1 family	1.05	1.34
F2D005	alcohol dehydrogenase	−0.84	−0.69
F2D017	Predicted protein	0.59	
F2D5K9	Predicted protein belonging to the “GDSL” lipolytic enzyme family	0.63	0.69
F2DE84	Predicted protein	0.75	0.65
F2DMQ8	Predicted protein belonging to the “GDSL” lipolytic enzyme family	2.95	0.86
F2DV72	Maltase	2.51	0.90
F2E8R9	Predicted protein belonging to the short-chain dehydrogenases/reductases (SDR) family	−1.01	
F2EAZ9	Predicted protein	0.85	
F2EFX9	Predicted protein	1.24	
Q5UNP2	Non-specific lipid-transfer protein	−0.68	
M0UYT8	α-galactosidase	1.03	
M0WAL0	Phytocyanin domain-containing protein	−0.82	
F2ELX8	Chitinase	1.41	
M0Z1D4	GDSL esterase/lipase	0.79	0.82
M0Z4H7	SCP domain-containing protein	1.83	1.66
M0XPC4	Uncharacterized protein belonging to the peptidase C1 family	−0.69	−0.75
M0YKC6	Uncharacterized protein belonging to the expansin family		−0.82
M0YQS0	Uncharacterized protein (Belongs to the expansin family. Expansin B subfamily)		−0.99
M0YV20	mannan endo-1,4-beta-mannosidase		1.45
F2DMG9	Predicted protein		0.67
B4ESF5	Papain-like cysteine proteinase		1.10
F2D8G6	Predicted protein		0.60
F2E3V0	ATP-dependent 6-phosphofructokinase	−0.63	
M0X1Y4	Aldose 1-epimerase	0.62	0.64
F2E008	Aldose 1-epimerase		0.59
F2DEG9	Phosphoenolpyruvate carboxykinase (ATP)		−0.59
F2DY58	Beta-amylase (Fragment)		−0.75
A0A8I7BF21	α-amylase		1.51
F2ECM4	Predicted protein belonging to the peptidase A1 family	0.62	
F2EDB2	Predicted protein	0.66	
F2EGD3	Predicted protein	0.81	
M0X1N3	Phospholipase D	−0.88	
M0W099	Thaumatin-like protein	1.45	
A3RHE5	Dehydrin Rab16C	−1.23	−0.61
F2CUJ9	Predicted protein	−0.62	
F2DJA3	Predicted protein	−0.95	
F2E5X4	Predicted protein belonging to the cystatin family. Phytocystatin subfamily	1.06	
F2E9C4	Predicted protein belonging to the LEA type 4 family	−1.09	−0.74
F2EBU5	Predicted protein belonging to the MIP/aquaporin (TC 1.A.8) family	−0.62	
F2EKR2	Predicted protein belonging to the caleosin family	−1.36	−1.04
F2ELE6	Glutathione reductase	−0.63	
Q0GA59	Dehydrin	−0.99	−1.07
Q7XZK3	Thioredoxin	−0.64	
M0WI75	Water stress and hypersensitive response domain-containing protein	−0.86	
M0Y565	Uncharacterized protein belonging to the LEA type 1 family	−0.83	
F2DTH1	Predicted protein belonging to the glycosyl hydrolase 17 family		1.41
F2DZW3	Predicted protein		−1.34
F2E8C4	Predicted protein belonging to the peptidase A1 family		0.60
F2EAF6	Predicted protein		0.68
F2D4M8	Predicted protein		1.06
F2DG27	Predicted protein		−0.61
F2DJM5	Predicted protein		−0.69
M0VYA0	Non-specific lipid-transfer protein		−1.28
F2CSC9	Predicted protein		−1.09
F2EI71	Predicted protein belonging to the DEFL family		−1.01
F2EKV9	Germin-like protein		0.59

## Data Availability

The original contributions presented in the study are included in the article/Supplementary Materials, further inquiries can be directed to the corresponding author.

## Supplementary Materials

The following supporting information can be downloaded at: https://www.mdpi.com/article/10.3390/metabo16040250/s1, Figure S1: Significant variability in changes in β-glucan content during germination of barley seeds.; Figure S2: Pearson’s correlation coefficients of all samples; Figure S3: Volcano plots showing differential protein expression; Figure S4: Distribution of the number of GO terms in the comparison of the three time periods; Figure S5: KEGG pathway analysis broad category distribution map; Figure S6: Relevant proteins involved in the β-glucan degradation pathway in B.vs.A was verified by real-time quantitative PCR analysis (qPCR); Figure S7. Protein–protein interaction (PPI) networks of differentially expressed proteins (DEPs) in the (A) B vs. A, (B) C vs. B, and (C) C vs. A comparisons. Table S1: Sequence of gene primers for qPCR assay; Table S2: List of peptide identifications; Table S3: Differently expressed proteins; Table S4: summary; Table S5: GO annotations in the three comparison groups; Table S6: GO terminology; Table S7: KEGG pathway analysis results for the three comparison groups (B vs. A, C vs. B, and C vs. A); Table S8: Mascot search engine parameters used for protein identification; Table S9: Protein–protein interaction (PPI) network data for the C vs. A comparison group. Tables S7–S9 contain additional supporting data (KEGG pathways, Mascot parameters, and PPI network for the third group) that are not directly cited in the main text but are provided as supplementary information for readers interested in the full dataset.

## References

[1] Visioni A., Basile B., Amri A., Sanchez-Garcia M., Corrado G. (2023). Advancing the Conservation and Utilization of Barley Genetic Resources: Insights into Germplasm Management and Breeding for Sustainable Agriculture. Plants.

[2] Finocchiaro F., Terzi V., Delbono S. (2023). Barley: From Molecular Basis of Quality to Advanced Genomics-Based Breeding. Compendium of Crop Genome Designing for Nutraceuticals.

[3] Tricase C., Amicarelli V., Lamonaca E., Rana R.L. (2018). Economic Analysis of the Barley Market and Related Uses. Grasses as Food and Feed.

[4] Sakellariou M., Mylona P.V. (2020). New uses for traditional crops: The case of barley biofortification. Agronomy.

[5] Garcia-Gimenez G., Russell J., Aubert M.K., Fincher G.B., Burton R.A., Waugh R., Tucker M.R., Houston K. (2019). Barley grain (1,3;1,4)-β-glucan content: Effects of transcript and sequence variation in genes encoding the corresponding synthase and endohydrolase enzymes. Sci. Rep..

[6] Walling J.G., Sallam A.H., Steffenson B.J., Henson C., Vinje M.A., Mahalingam R. (2022). Quantitative trait loci impacting grain β-glucan content in wild barley (*Hordeum vulgare* ssp. spontaneum) reveal genes associated with cell wall modification and carbohydrate metabolism. Crop Sci..

[7] Chen H., Nie Q., Xie M., Yao H., Zhang K., Yin J., Nie S. (2019). Protective effects of β-glucan isolated from highland barley on ethanol-induced gastric damage in rats and its benefits to mice gut conditions. Food Res. Int..

[8] Zhang G.Q., Zhang G.P., Zeng X.Q., Xu Q.J., Wang Y.L., Yuan H.J., Zhang Y.H., Nyima T. (2020). Quantitative proteome profiling provides insight into the proteins associated with β-Glucan accumulation in hull-less barley grains. J. Agric. Food Chem..

[9] QuZhen N., Namgyal L., Dondrup D., Wang Y., Wang Z., Cai X.-X., Lu B.-R., Qiong L. (2024). Abundant Genetic Diversity Harbored by Traditional Naked Barley Varieties on Tibetan Plateau: Implications in Their Effective Conservation and Utilization. Biology.

[10] Mio K., Ogawa R., Tadenuma N., Aoe S. (2022). Arabinoxylan as well as β-glucan in barley promotes GLP-1 secretion by increasing short-chain fatty acids production. BB Rep..

[11] Geng L., He X., Ye L., Zhang G. (2022). Identification of the genes associated with β-glucan synthesis and accumulation during grain development in barley. Food Chem..

[12] Cai G., Kim S.C., Li J., Zhou Y., Wang X. (2020). Transcriptional regulation of lipid catabolism during seedling establishment. Mol. Plant.

[13] Kaur M., Tak Y., Bhatia S., Asthir B., Lorenzo J.M., Amarowicz R. (2021). Crosstalk during the carbon–nitrogen cycle that interlinks the biosynthesis, mobilization and accumulation of seed storage reserves. Int. J. Mol. Sci..

[14] Islam M.Z., An H.G., Kang S.J., Lee Y.T. (2021). Physicochemical and bioactive properties of a high β-glucan barley variety ‘Betaone’ affected by germination processing. Int. J. Biol. Macromol..

[15] Liu S., Wang W., Lu H., Shu Q., Zhang Y., Chen Q. (2022). New perspectives on physiological, biochemical and bioactive components during germination of edible seeds: A review. Trends Food Sci. Technol..

[16] Marconi O., Tomasi I., Dionisio L., Perretti G., Fantozzi P. (2014). Effects of malting on molecular weight distribution and content of water-extractable β-glucans in barley. Food Res. Int..

[17] Burton R.A., Collins H.M., Kibble N.A., Smith J.A., Shirley N.J., Jobling S.A., Henderson M., Singh R.R., Pettolino F., Wilson S.M. (2011). Over-expression of specific HvCslF cellulose synthase-like genes in transgenic barley increases the levels of cell wall (1,3;1,4)-β-d-glucans and alters their fine structure. Plant Biotechnol. J..

[18] Burton R.A., Wilson S.M., Hrmova M., Harvey A.J., Shirley N.J., Medhurst A., Stone B.A., Newbigin E.J., Bacic A., Fincher G.B. (2006). Cellulose synthase-like CslF genes mediate the synthesis of cell wall (1,3;1,4)-β-D-glucans. Science.

[19] Schreiber M., Wright F., MacKenzie K., Hedley P.E., Schwerdt J.G., Little A., Burton R.A., Fincher G.B., Marshall D., Waugh R. (2014). The barley genome sequence assembly reveals three additional members of the CslF (1,3;1,4)-β-glucan synthase gene family. PLoS ONE.

[20] Houston K., Russell J., Schreiber M., Halpin C., Oakey H., Washington J.M., Booth A., Shirley N., Burton R.A., Fincher G.B. (2014). A genome wide association scan for (1,3;1,4)-β-glucan content in the grain of contemporary 2-row Spring and Winter barleys. BMC Genom..

[21] García-Castro A., Guzmán Ortiz F.A., Hernández G., Román-Gutiérrez A.D. (2024). Analysis of bioactive compounds in lyophilized aqueous extracts of barley sprouts. J. Food Meas. Charact..

[22] García-Castro A., Román-Gutiérrez A.D., Guzmán-Ortiz F.A., Cariño-Cortés R. (2024). Antihypertensive Effect of Perla and Esmeralda Barley (*Hordeum vulgare* L.) Sprouts in an Induction Model with L-NAME In Vivo. Metabolites.

[23] Zhu L., Wang Z., Gao L., Chen X. (2024). Unraveling the Potential of γ-Aminobutyric Acid: Insights into Its Biosynthesis and Biotechnological Applications. Nutrients.

[24] Wiśniewski J.R., Zougman A., Nagaraj N., Mann M. (2009). Universal sample preparation method for proteome analysis. Nat. Methods.

[25] Batth T.S., Francavilla C., Olsen J.V. (2014). Off-line high-pH reversed-phase fractionation for in-depth phosphoproteomics. J. Proteome Res..

[26] Sherman B.T., Hao M., Qiu J., Jiao X., Baseler M.W., Lane H.C., Imamichi T., Chang W. (2022). DAVID: A web server for functional enrichment analysis and functional annotation of gene lists (2021 update). Nucleic Acids Res..

[27] Binns D., Dimmer E., Huntley R.P., Barrell D., O'Donovan C., Apweiler R. (2009). QuickGO: A web-based tool for Gene Ontology searching. Bioinformatics.

[28] Kanehisa M., Furumichi M., Sato Y., Kawashima M., Ishiguro-Watanabe M. (2022). KEGG for taxonomy-based analysis of pathways and genomes. Nucleic Acids Res..

[29] Szklarczyk D., Kirsch R., Koutrouli M., Nastou K., Mehryary F., Hachilif R., Gable A.L., Fang T., Doncheva N.T., Pyysalo S. (2022). The STRING database in 2023: Protein–protein association networks and functional enrichment analyses for any sequenced genome of interest. Nucleic Acids Res..

[30] Franz M., Lopes C., Fong D., Kucera M., Cheung M., Siper M.C., Huck G., Dong Y., Sumer O., Bader G.D. (2023). Cytoscape.js 2023 update: A graph theory library for visualization and analysis. Bioinformatics.

[31] Wilson S.M., Burton R.A., Collins H.M., Doblin M.S., Pettolino F.A., Shirley N., Fincher G.B., Bacic A. (2012). Pattern of deposition of cell wall polysaccharides and transcript abundance of related cell wall synthesis genes during differentiation in barley endosperm. Plant Physiol..

[32] Han F., Ullrich S.E., Chirat S., Menteur S., Jestin L., Sarrafi A., Hayes P.M., Jones B.L., Blake T.K., Wesenberg D.A. (1995). Mapping of β-glucan content and β-glucanase activity loci in barley grain and malt. Theor. Appl. Genet..

[33] Kanehisa M., Goto S. (2000). KEGG: Kyoto encyclopedia of genes and genomes. Nucleic Acids Res..

[34] Liang K., Liang S., Zhu H. (2020). Comparative proteomics analysis of the effect of selenium treatment on the quality of foxtail millet. LWT.

[35] Liu C., Yang F., Li L., Han X., Chen H., Sha A., Jiao C. (2024). Isobaric Tags for Relative and Absolute Quantitation-Based Proteomics Analysis Revealed Proteins Involved in Drought Response during the Germination Stage in Faba Bean. Metabolites.

[36] Nadiminti P.P., Wilson S.M., van de Meene A., Hao A., Humphries J., Ratcliffe J., Yi C., Peirats-Llobet M., Lewsey M.G., Whelan J. (2023). Spatiotemporal deposition of cell wall polysaccharides in oat endosperm during grain development. Plant Physiol..

[37] Francin-Allami M., Bouder A., Geairon A., Alvarado C., Le-Bot L., Daniel S., Shao M.Q., Laudencia-Chingcuanco D., Vogel J.P., Guillon F. (2023). Mixed-linkage glucan is the main carbohydrate source and starch is an alternative source during Brachypodium grain germination. Int. J. Mol. Sci..

[38] Song J.Q., Liu X.J., Li X.X., Wang H.F., Chu R.W., Qu F.F., Zhang S.X., Li Q.L. (2022). Transcriptome analysis reveals genes and pathways associated with salt tolerance during seed germination in Suaeda liaotungensis. Int. J. Mol. Sci..

[39] Voiniciuc C. (2022). Modern mannan: A hemicellulose’s journey. New Phytol..

[40] Opassiri R., Pomthong B., Onkoksoong T., Akiyama T., Esen A., Ketudat Cairns J.R. (2006). Analysis of rice glycosyl hydrolase family 1 and expression of Os4bglu12 β-glucosidase. BMC Plant Biol..

[41] Zang X., Liu M., Fan Y., Xu J., Xu X., Li H. (2018). The structural and functional contributions of β-glucosidase-producing microbial communities to cellulose degradation in composting. Biotechnol. Biofuels.

[42] Höftberger M., Althammer M., Foissner I., Tenhaken R. (2022). Galactose induces formation of cell wall stubs and cell death in Arabidopsis roots. Planta.

[43] Bhatia S., Singh A., Batra N., Singh J. (2020). Microbial production and biotechnological applications of α-galactosidase. Int. J. Biol. Macromol..

[44] Ronimus R.S., Morgan H.W. (2001). The biochemical properties and phylogenies of phosphofructokinases from extremophiles. Extremophiles.

[45] Poolman B., Royer T.J., Mainzer S.E., Schmidt B.F. (1990). Carbohydrate utilization in Streptococcus thermophilus: Characterization of the genes for aldose 1-epimerase (mutarotase) and UDPglucose 4-epimerase. J. Bacteriol..

[46] Sheshukova E.V., Komarova T.V., Pozdyshev D.V., Ershova N.M., Shindyapina A.V., Tashlitsky V.N., Sheval E.V., Dorokhov Y.L. (2017). The intergenic interplay between aldose 1-epimerase-like protein and pectin methylesterase in abiotic and biotic stress control. Front. Plant Sci..

[47] Andriotis V.M.E., Rejzek M., Barclay E., Rugen M.D., Field R.A., Smith A.M. (2016). Cell wall degradation is required for normal starch mobilisation in barley endosperm. Sci. Rep..

[48] Janeček Š., Svensson B., MacGregor E.A. (2014). α-Amylase: An enzyme specificity found in various families of glycoside hydrolases. Cell. Mol. Life Sci..

[49] Han C., Yang P. (2015). Studies on the molecular mechanisms of seed germination. Proteomics.

[50] Kaplan F., Guy C.L. (2004). β-Amylase induction and the protective role of maltose during temperature shock. Plant Physiol..

[51] Verma V., Ravindran P., Kumar P.P. (2016). Plant hormone-mediated regulation of stress responses. BMC Plant Biol..

[52] Han N., Na C., Chai Y., Chen J., Zhang Z., Bai B., Bian H., Zhang Y., Zhu M. (2016). Over-expression of (1,3;1,4)-β-D-glucanase isoenzyme EII gene results in decreased (1,3;1,4)-β-D-glucan content and increased starch level in barley grains. J. Sci. Food Agric..

[53] Aoki H., Seki M., Nakata M., Nakano Y., Nagamine T. (2021). Effects of starch synthase IIIa (ssIIIa) genotype and grain hardness on the barley β-glucan content and grain morphology traits. Breed. Res..

[54] Riseh R.S., Vazvani M.G., Kennedy J.F. (2023). β-Glucan-induced disease resistance in plants: A review. Int. J. Biol. Macromol..

[55] Ikuta S., Fukusaki E., Shimma S. (2022). Visualization of Glutamate Decarboxylase Activity in Barley Seeds under Salinity Stress Using Mass Microscope. Metabolites.

[56] Jeandet P., Formela-Luboińska M., Labudda M., Morkunas I. (2022). The role of sugars in plant responses to stress and their regulatory function during development. Int. J. Mol. Sci..

[57] Dermendjiev G., Schnurer M., Weiszmann J., Wilfinger S., Ott E., Gebert C., Weckwerth W., Ibl V. (2021). Tissue-Specific Proteome and Subcellular Microscopic Analyses Reveal the Effect of High Salt Concentration on Actin Cytoskeleton and Vacuolization in Aleurone Cells during Early Germination of Barley. Int. J. Mol. Sci..

[58] Bacete L., Mélida H., Miedes E., Molina A. (2017). Plant cell wall-mediated immunity: Cell wall changes trigger disease resistance responses. Plant J..

[59] Rebaque D., del Hierro I., López G., Bacete L., Vilaplana F., Dallabernardina P., Pfrengle F., Jordá L., Sánchez-Vallet A., Pérez R. (2021). Cell wall-derived mixed-linked β-1,3/1,4-glucans trigger immune responses and disease resistance in plants. Plant J..

[60] Barghahn S., Arnal G., Jain N., Petutschnig E., Brumer H., Lipka V. (2021). Mixed Linkage β-1,3/1,4-Glucan Oligosaccharides Induce Defense Responses in *Hordeum vulgare* and *Arabidopsis thaliana*. Front. Plant Sci..

[61] Yin Y., Hu M., Yang Z., Zhu J., Fang W. (2024). Salicylic acid promotes phenolic acid biosynthesis for the production of phenol acid-rich barley sprouts. J. Sci. Food Agric..

[62] Dong N.Q., Lin H.X. (2021). Contribution of phenylpropanoid metabolism to plant development and plant–environment interactions. J. Integr. Plant Biol..

[63] Zhu T., Fang X., Wei S., Liu Y., Han Y., Xie J., Ding Q., Ma L. (2019). Genome-wide identification, phylogeny and expression profiling of class III peroxidases gene family in Brachypodium distachyon. Gene.

[64] De Oliveira F.K., Santos L.O., Buffon J.G. (2021). Mechanism of action, sources, and application of peroxidases. Food Res. Int..

[65] Zhang H., Zhang X., Zhao J., Sun L., Wang H., Zhu Y., Xiao J., Wang X. (2021). Genome-Wide Identification of GDSL-Type Esterase/Lipase Gene Family in Dasypyrum villosum L. Reveals That DvGELP53 Is Related to BSMV Infection. Int. J. Mol. Sci..

[66] Chepyshko H., Lai C.P., Huang L.M., Liu J.H., Shaw J.F. (2012). Multifunctionality and diversity of GDSL esterase/lipase gene family in rice (*Oryza sativa* L. japonica) genome: New insights from bioinformatics analysis. BMC Genomics.

[67] Kumar M., Brar A., Yadav M., Chawade A., Vivekanand V., Pareek N. (2018). Chitinases-potential candidates for enhanced plant resistance towards fungal pathogens. Agriculture.

[68] Jensen L.G. (2004). Developmental Patterns of Enzymes and Proteins During Mobilization of Endosperm Stores in Germinating Barley Grains. Hereditas.

[69] Xu D., Duan X., Wang B., Hong B., Ho T., Wu R. (1996). Expression of a late embryogenesis abundant protein gene, hva1, from barley confers tolerance to water deficit and salt stress in transgenic rice. Plant Physiol..

[70] Misas-Villamil J.C., van der Hoorn R.A., Doehlemann G. (2016). Papain-like cysteine proteases as hubs in plant immunity. New Phytol..

[71] Martínez M., Cambra I., Carrillo L., Diaz-Mendoza M., Diaz I. (2009). Characterization of the entire cystatin gene family in barley and their target cathepsin L-like cysteine-proteases, partners in the hordein mobilization during seed germination. Plant Physiol..

