# Supplementary material for: TMT Proteomics-Based Study of Proteins and Pathways Associated with β-Glucan Degradation in Barley Germination
Source: Metabolites. 2026 Apr 7;16(4):250. doi: 10.3390/metabo16040250 (PMC13117924; doi:10.3390/metabo16040250)
Supplement: Supplementary file 1 [file metabolites-16-00250-s001.zip › Supplementary figures.pdf]

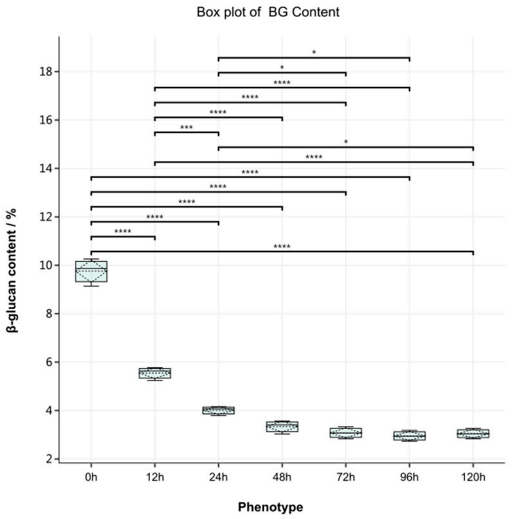

Figure S1. Significant variability in changes in β-glucan content during germination of barley seeds.

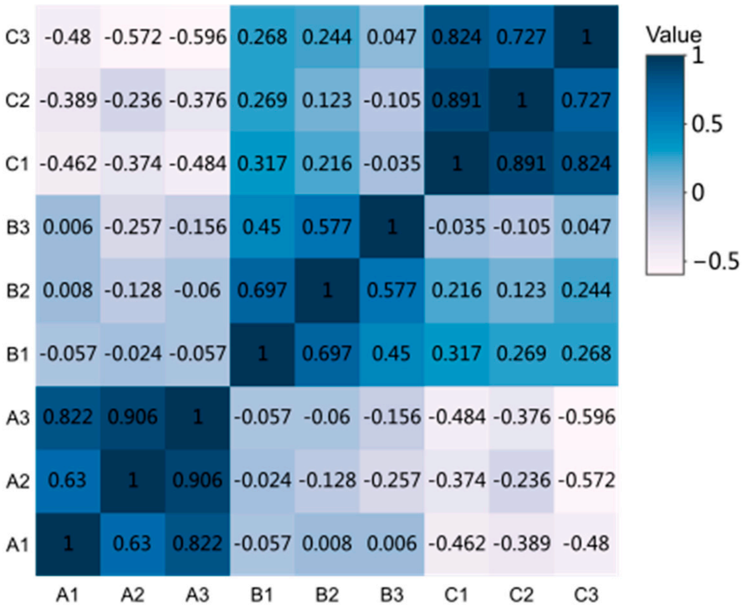

Figure S2. Pearson's correlation coefficients of all samples.

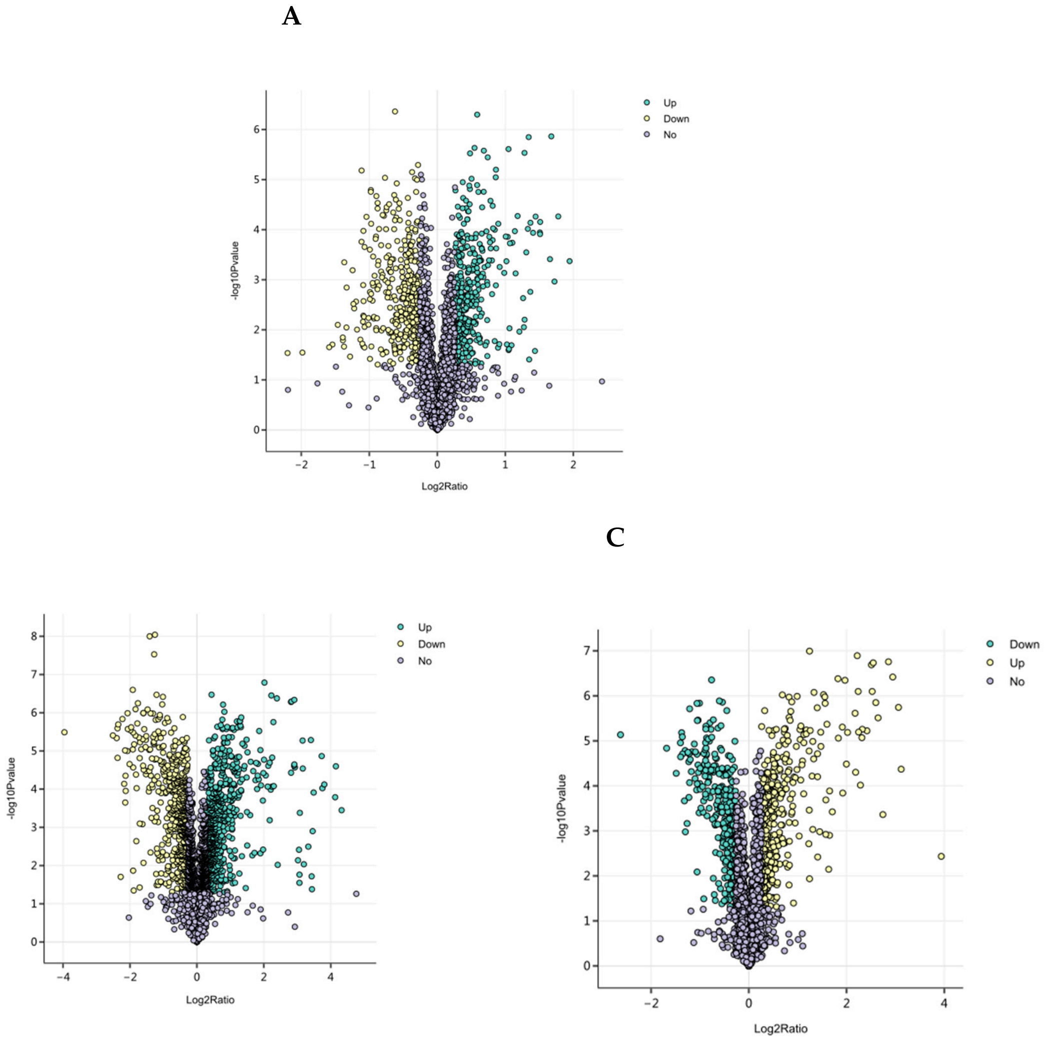

Figure S3. Volcano plots showing differential protein expression. (A) B.VS.A Comparison of differential protein volcano distribution maps. (B) C.VS.B Comparison of differential protein volcano distribution maps. (C) C.vs.A Comparison of differential protein volcano distribution maps.

https://doi.org/10.3390/xxxxx

A

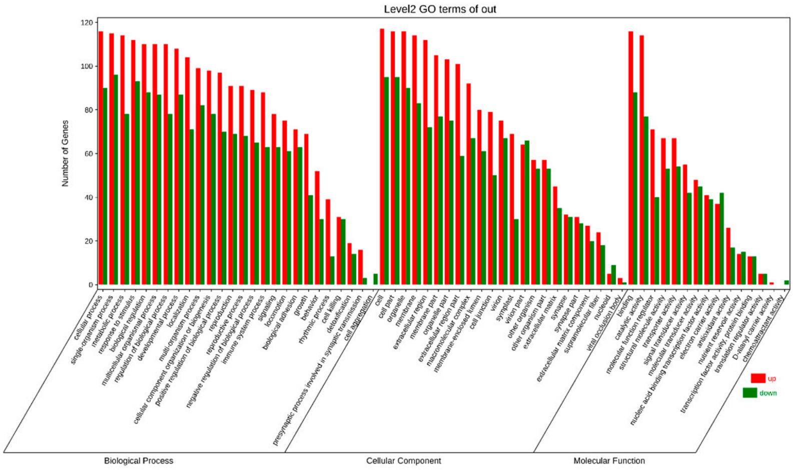

B

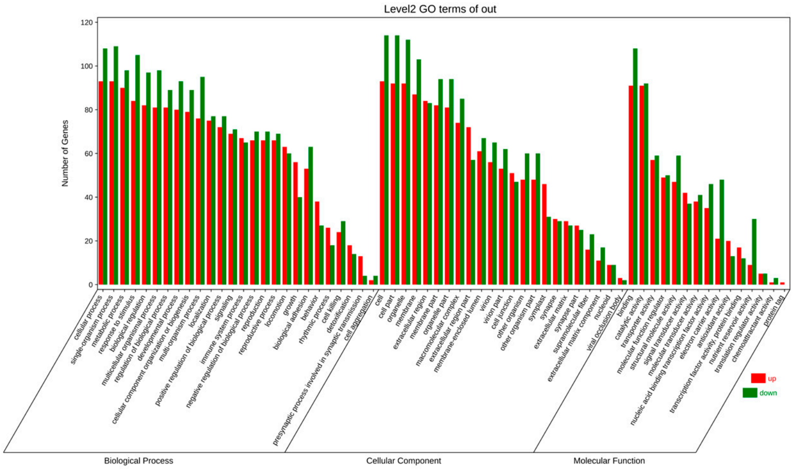

C

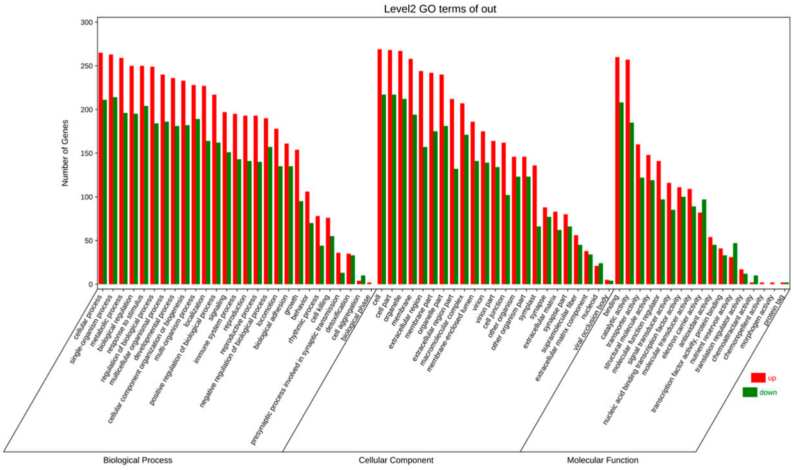

Figure S4. Distribution of the number of GO terms in the comparison of the three time periods. (A) B.VS.A. (B) C.VS.B. (C) C.vs.A. Red for upward adjustments, green for downward adjustments.

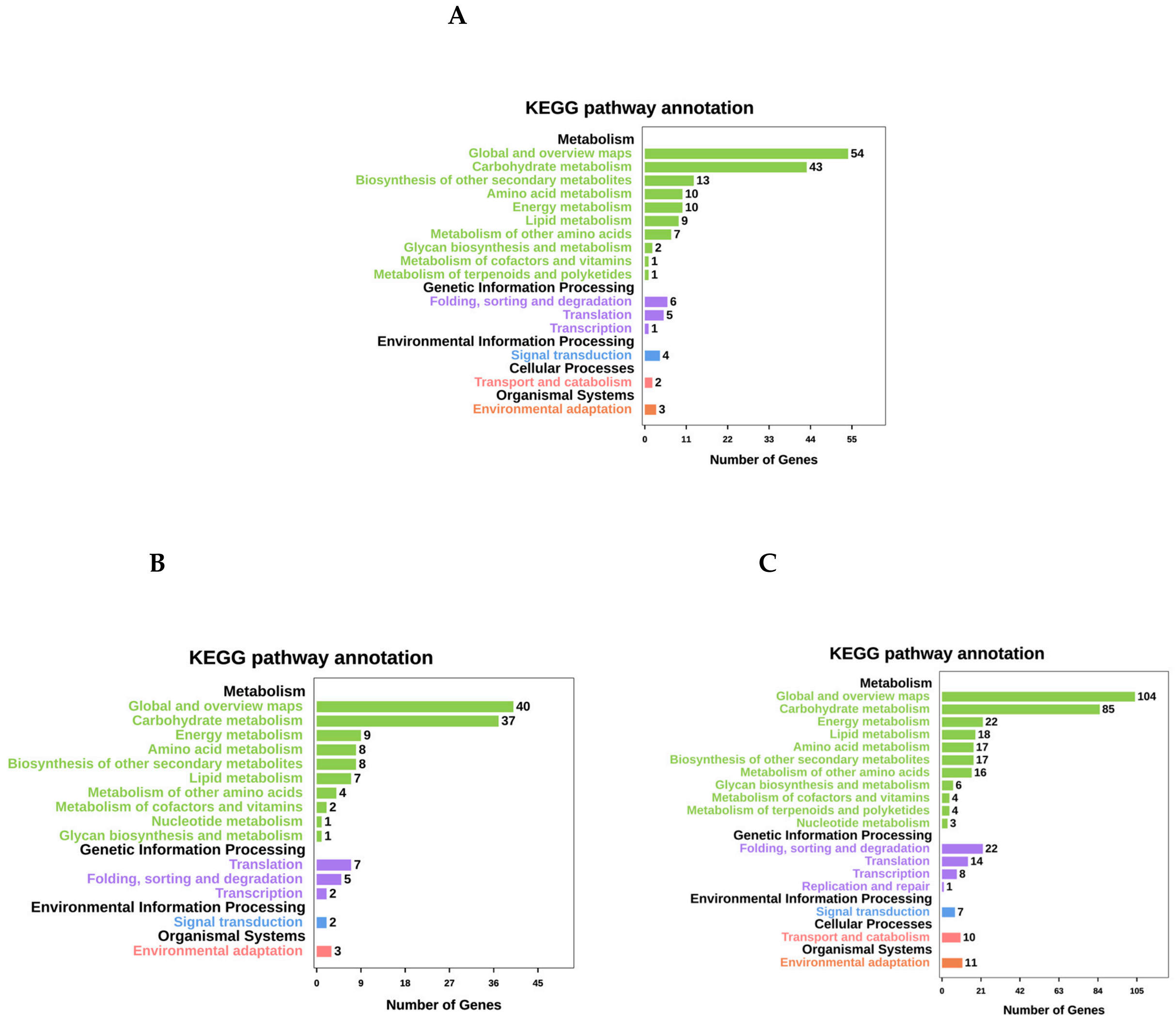

Figure S5. KEGG pathway analysis broad category distribution map. (A) B.VS.A. (B) C.VS.B. (C) C.vs.A.

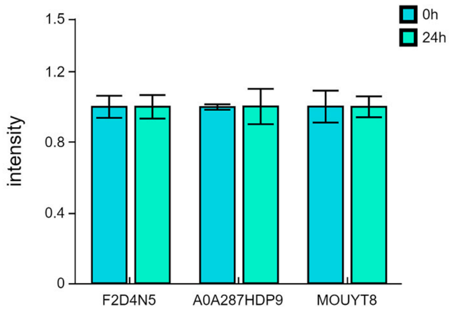

Figure S6. Relevant proteins involved in the β-glucan degradation pathway in B.vs.A was verified by real-time quantitative PCR analysis (qPCR). All data were expressed as mean ± SD (n = 3).

A

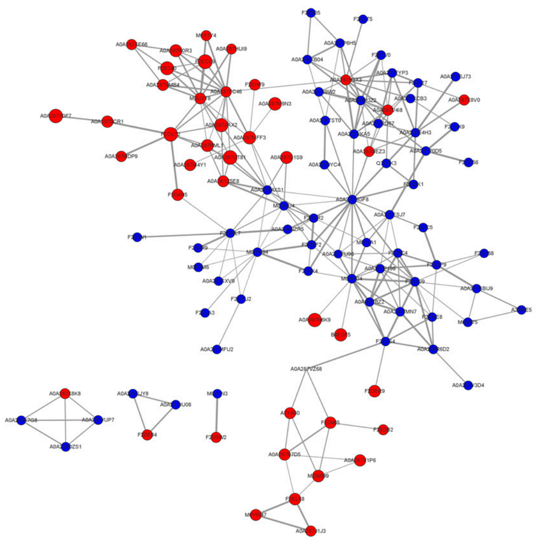

B

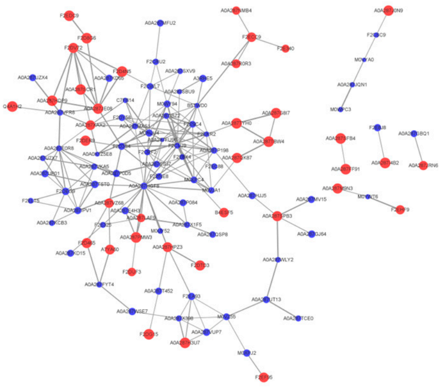

C

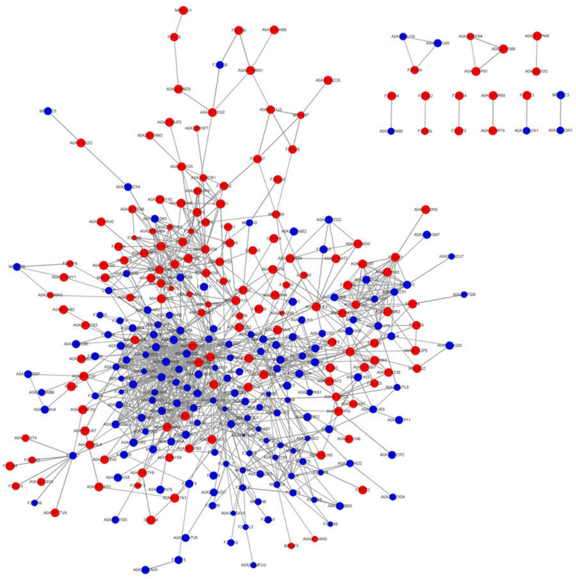

Figure S7. Protein–protein interaction (PPI) networks of differentially expressed proteins (DEPs) in the (A) B vs. A, (B) C vs. B, and (C) C vs. A comparisons. Red and blue nodes represent up-regulated and down-regulated DEPs, respectively.
